# Egyptian patients’/guardians’ experiences and perception about clinical informed consent and its purpose: Cross sectional study

**DOI:** 10.1371/journal.pone.0252996

**Published:** 2021-06-14

**Authors:** Ammal M. Metwally, Hala A. Amer, Hend I. Salama, Safaa I. Abd El Hady, Raefa R. Alam, Ahmed Aboulghate, Hanan A. Mohamed, Hanan M. Badran, Amal A. Saadallah, Marwa M. El-Sonbaty, Eman Eltahlawy, Walaa Saad, Amira Mohsen, Ghada A. Abdel-Latif, Asmaa M. Fathy, Amal I. Hassanain, Abdelmoneim Eldali

**Affiliations:** 1 Medical Research Division, Community Medicine Research Department, National Research Centre, Dokki, Cairo, Egypt; 2 Department of Infection Control, King Saud Medical City, Riyadh, KSA; 3 National Blood Transfusion Services Mansoura Region, Ministry of Health and Population, Egypt; 4 Neuropsychiatry Dept., Ministry of Health and Population, Egypt; 5 Faculty of Nursing, Mansoura University, Mansoura, Egypt; 6 Egyptian Liver Research Institute And Hospital (ELRIAH), Mansoura, Egypt; 7 King Faisal Specialist Hospital & Research Centre, Riyadh, Saudi Arabia; 8 Medical Research Division, Child Health Department, National Research Centre, Cairo, Egypt; 9 Department of Pediatrics, College of Medicine, Taibah University, Madinah, K.S.A; 10 Environmental Research Division, Environmental Health and Occupational Medicine, National Research Centre, Egypt; 11 Department of biological anthropology, National Research Centre, Egypt, Cairo, Egypt; 12 Free-Lancer, Khartoum, Sudan; University of Haifa, ISRAEL

## Abstract

**Background:**

Informed consent (IC) is a healthcare standard emphasizing the meaning of human dignity as clarified in the Universal Declaration of Human Rights. Data about IC practices in Egypt is insufficient. This study aimed to assess the Egyptian patients’/guardians’ experiences about IC and their expectations about its practices’ purposes in general and according to the type of the healthcare facility.

**Methods:**

Self-administered questionnaire was carried out for 1092 participants who had undergone or were scheduled to a procedure requiring an IC at three studied types for Egyptian health care facilities. Ten statements were ranked twice by the participants to reflect their perception of IC purpose as per what is currently practiced and what they believe should be practiced.

**Results:**

IC implementation varies significantly (p<0.05) across the health care facilities in Egypt. The percentage of its implementation at the non-governmental facilities, governmental facilities, and university hospital was 85.9%, 77.8%, and 63.8 respectively. The first three ranked purposes of the current IC practices were: “Helping patient/guardian decide (64.9%)”, “Documenting patient’s/guardian’s decision (59.3%)”, and “Having shared decision (57.3%)”. The perceived purposes of IC to be practiced were: “Informing the patient/guardian (68.4%)”, “Making sure patient/guardian understand (65.3%)” and “Documenting patients/guardians decisions (65.1%)”. “Being a meaningless routine” was reported by the majority to be ranked as a low purpose for IC current and preferred practices.

**Conclusion:**

The practice of IC is common within the Egyptian medical community. Participants believe that information disclosure “Making sure patients understand” has to help in IC decision making and its main purpose. However, unfortunately, this is not perceived as a current purpose of IC. There was consensus agreement that documenting the patient’s/guardian’s decision and informing the patient/guardian are perceived as both important current and preferred purposes for IC practices.

## Introduction

Informed consent (IC) is a fundamental principle of health care. It is the process whereby the patient/ guardian and the health care practitioner engage in a dialogue about a proposed medical treatment nature, consequences, harms, benefits, risks, and alternatives [1]. Through this process, the health care provider discloses appropriate information to a competent patient/guardian so that a voluntary choice to accept or refuse treatment could be made [2].

The origin of the IC was dated back to ancient Egyptian times when doctors believed that their medicine had to benefit their patients while not being detrimental [3]. Furthermore, historical background about the use of formal informed consent for surgery dates back to the 17th century when it was used in the Islamic/Arabic culture [4]. It originates from the legal and ethical right indicating that the patient has to decide what happens to his/ her body and that the ethical duty of the physician is to involve the patient in his/her health care [5]. In the 1970s, IC was embraced as a correction to paternalism. Meanwhile, between the 1980s and 1990s, shared decision-making was considered as a necessary correction of “exaggerated individualism” [6]. Accordingly, the IC process is linked to the global target that has been set by the United Nation for sustainable development; goal no 3 for ensuring healthy lives and promoting wellbeing for all ages, and goal no 10 for reducing inequalities between and among countries” [7].

The current concept of IC developed on a principle which states that the fundamental element of the doctor-patient relationship is based on the patient’s free choice to undergo the proposed medical procedure [7]. The purpose of the informed consent process ranges from being routine paperwork to ideally enabling patients’ self-decision-making. It has been perceived by some patients and clinicians as mean to fulfill the social requirement of giving more authority to the customers in health care. Philosophically, IC is founded on the principle of respect for persons, which includes not only respect for autonomy but also liberty and wellbeing [8].

The quality of the IC in clinical practice is influenced by many factors [9,10]. These factors could be sociodemographic; such as the educational level or patient age affecting the process and result of IC [11,12]. Hence, parents or caregivers recognized as the appropriate ethical and legal surrogate medical decision-makers for their children and adolescents. This recognition affirms parents’ familiar understanding of their children’s affairs and respects family autonomy [13].

In Egypt, IC and sharing information are among the mandated Egyptian patients’ rights since 2005 [14] along with other rights such as access to health care [15–18] in general and specifically for reproductive rights [19,20], choice of care for communicable and non-communicable diseases [21–23], right for breastfeeding [24], school feeding [25], health education [26], participation in the treatment plan, confidentiality and privacy [27,28]. Moreover, the rights to donate organs after death [29].

There is however a lack of information about the practice of informed consent provision in Egyptian society and the individuals’ expectations about its role. This study aimed at identifying participants’ perception of IC purpose as per what is currently practiced and as per their preferences and comparing the current versus the preferred purposes of IC practices within and between types of health care facilities in Egypt.

The outcome of this research is expected to enlighten the health care policymakers about any required modification of the current informed consent process to fit in patients’ rights.

## Materials and methods

A Multi-center cross-sectional exploratory research was conducted in three different health care settings of Egypt during the period June 2017 to May 2018. The study facilities included governmental organizations affiliated with the Ministry of Health and Population in Egypt, a non-governmental private organization, and a teaching hospital which is named respectively; Health units and centers at Dekarness district of El Dakahlia Governorate, The Egyptian Liver institute and Hospital in El Dakahlia Governorate and El Mansoura university hospital. The facilities were selected based on accessibility, geographical coverage, and diversity of attending patients. Accordingly, the sample type of this study was considered representative of a larger population who undergo the same criteria of the studied facilities.

### Data collection and analysis

Participants were randomly selected from adult patients and parents of ill children and adolescents who had undergone medical or surgical procedures requiring specific written informed consent over the previous three months or were scheduled to undergo one within the following three months. The study targeted both males and females in the age range 20 to 70 years with low socioeconomic standards and low scholarly level and those who were exposed to either elective or emergency procedures.

**Fig 1:**
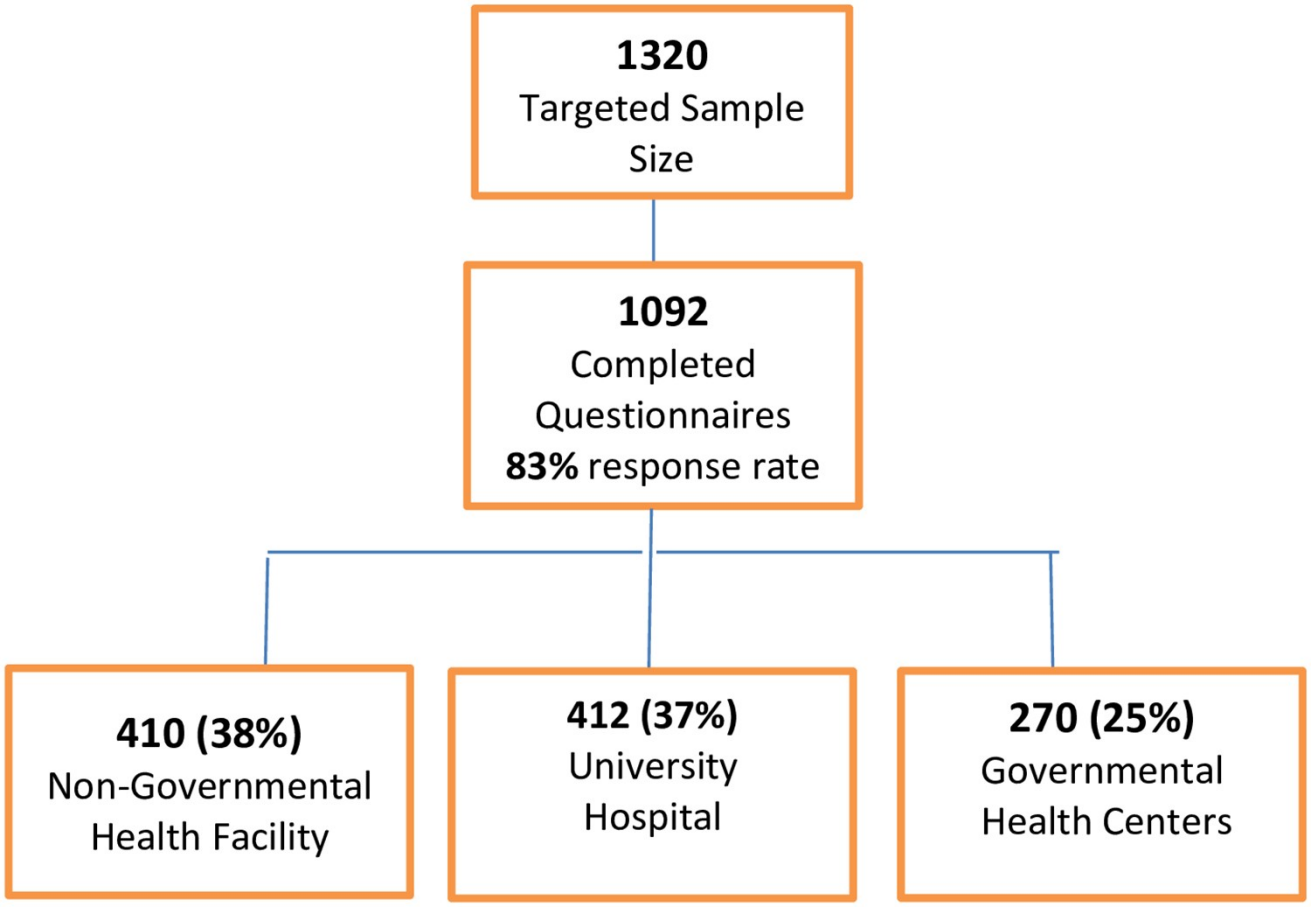
Response rate of the participants and their distribution among the study facilities.

Sample size calculation was based on the following assumption per setting: a two-sided 95% confidence interval with a width equal to 0.100, sample proportion of 0.5 assuming that 50% are informed to provide the maximum width for a confidence interval with the calculated sample size. Accordingly, the calculated sample size was equal to 402 per set. On adding 10% expected losses, a sample size of 440 participants per setting was targeted to ensure accuracy of the data with a total sample of 1092 who completed the questionnaire [30]. The response rate among the study facilities are presented in Fig 1 (attachment).

The self-administrated anonymous questionnaire used was a reviewed version adapted from Hammami [31] with the author’s permission. Few edits had been done to Hammami version to be convenient with the present study structure after conducting a pilot study for 30 participants (10 per each facility). Questions concerning participants’ sociodemographic data were added. Besides, due to the inclusion of ill children’s guardians, the revised questionnaire included modification to some questions accordingly.

Participants received an explanation and gave their verbal consent to participate. Verbal consent was taken because of the following considerations; the survey was anonymous, no invasive procedures were included, and no risk was expected and most importantly. Therefore, the Institutional Medical Research Ethics Committee at the National Research Centre of Egypt approved consent as verbal. All participants were identified by a serial number and the information at the individual level was kept strictly confidential.

Data were collected about participants’ demographic characteristics, procedure type, and IC process. The questionnaire consisted of two parts each of ten statements about the potential purpose of the informed consent process which were presented to participants in random order. One part was considering participants’ perception of the current purpose at the study institutions and the other part about their perception of the preferred purpose. Participants were then instructed to rank the ten statements in each part on a ten-point scale in which 1 was “most reflective” and 10 was “least reflective”. The ten statements abbreviations are listed in Table 3.

Data were analyzed using SPSS version 20. The grouping by the three health care facilities applied to tackle the confounding problems. *Kruskal-Wallis and Wilcoxon Signed ranks tests were used to compare statement ranking. Pearson’s Chi-square test (χ^2^) was used to detect whether there was any significant difference between data presented by numbers and percentage. The level of significance used was 95% with alpha = 5%.

### Ethical approval

The study was approved by the Medical Research Ethics Committee of the National Research Centre of Egypt (Ethical approval number: 16051). The conduct of the study complied with the International Ethical Guidelines for Biomedical Research Involving Human Subjects [32].

## Results

Out of the 1092 participants who filled in the questionnaire, 48.3% of them were males with the highest males’ participation occurring at the non-government hospital (57.6%) and the highest females’ participation occurring at the university hospital (62.4%). Almost nine in every ten of the participants were married. Considering the type of procedure undergone, elective procedures were significantly higher than emergency procedures, especially in the non-governmental hospital (82%) with a highly statistically significant difference (p<0.001) [Table 1].

**Table 1:** Socio-demographic characteristics of the study participants according to the type of the healthcare facility.

	Governmental 1ry Healthcare centers	Non- governmental hospital	University hospital
**No. of study participants**	270	410	412
**Age in years [mean** ± **SD]**	44.4±13.33	51.46±12.16	50.89±11.73
**Gender [no. (%)]**			
Male	136(50.4)	236(57.6)	155 (37.6)
Female	134(49.6)	174(42.4)	257 (62.4)
X^2^ <0.001, sig			
**Marital status [no. (%)]**			
Single	12(4.4)	15(3.7)	11(2.7)
Married	240(88.9)	365(89.0)	369(89.6)
Divorced	1(0.7)	7(1.7)	23(5.6)
Widow	17(5.9)	23(5.6)	9(2.2)
X^2^ <0.001, sig			
**Type of medical procedure [no. (%)]**			
Elective	174(64.4)	336(82.0)	257(62.4)
Emergency	96(35.6)	74(18.0)	155(37.8)
X^2^ <0.001, sig			
**Information provided [no. (%)]**			
Enough info	210(77.8)	352(85.9)	263(63.8)
Not enough info	60(22.2)	52(12.7)	84(20.4)
No info applied	0 (0.0)	6(1.5)	65(15.8)
X^2^ between provision of enough versus not enough info <0.001, sig			
**Type of consent [no. (%)]**			
Written consent	248 (91.9)	394(85.1)	250 (60.7)
Oral consent	14 (5.2)	54(13.2)	115(27.9)
No consent	8 (3)	7(1.7)	47(11.4)
X^2^ <0.001, sig			

no.: count.

Considering the type of informed consent provided to the patients, parents, or other caregivers, written consent was significantly the most common type in the primary healthcare center followed by the non-governmental hospital then by the university hospital (91.9%, 85.1%, and 60.7% respectively). More than two-thirds of the participants reported that “enough information” was provided to them during the informed consent process especially among the non-governmental hospital group (85.9%). “No information provided” was significantly higher among the university hospital group (15.8%) compared to the other two settings [Table 1]. It was also observed that for any procedures concerning children and/or adolescents, their guardians always talk on their behalf without considering any consultation with them. Guardians represented 45.79% of the participants (500 out of 1092), 204 (40.8%) from the governmental primary healthcare centers, 140 (28%) from the university hospital, 156 (31.2%) from the non-government hospital.

Receiving enough information during the IC process was reported by almost three-quarters (75.6%) of the interviewed participants. 85.4% of females participants reported significantly receiving little or no information during the IC process. When comparing the information received by place of the study, the least received information was significantly reported among those who were interviewed at the university hospital (55.8%) irrespective of the types of the medical procedures they were exposed to (p>0.05). Participants who reported receiving enough information were significantly found among those who were interviewed in the non-government hospitals (42.7%) [Table 2].

**Table 2:** Factors influencing participants’ perception of the amount of information received during the informed consent process.

	Enough information received [: 825 (75.6%)] Count (%)	Little or no information received [n:267 (24.4%)] Count (%)	p-value of X^2^
***Gender***			
Male	590(71.5)	39(14.6)	0.001*
Female	235(28.5)	228(85.4)	
***Place of the study***			
Governmental 1ry Health Care Centers	210 (25.5)	60 (22.5)	<0.001*
Non-Government Hospital	352 (42.7)	58 (21.7)	
University Hospital	263 (31.8)	149 (55.8)	
***Medical Procedure***:			
Elective	584 (70.8)	183(68.5)	0.77
Emergency	241 (29.2)	84 (31.5)	

n.: number of participant.

* Significant P-value.

The ten statements abbreviations were listed in [Table 3] showing the comparison between patients’ perceptions of current and preferred purposes of IIC practices according to the health facility type. There were significant differences concerning the majority of the perceived current as well as the preferred purposes of the IC practices between the studied health facilities except for three current and one preferred purposes. Consensus agreement was found between the three healthcare settings regarding three perceived current purposes of IC practices; in order for taking away compensation rights” followed by “Informing patients/guardians” and “Having shared decision”. Whereas, “for litigation protection” is considered the least preferred purpose by patients attending any of the studied health facility.

**Table 3:** Comparison between patients’ perceptions of the current and preferred purposes of the IC practices between the study facilities.

	Current Practice Mean± SD Median (25%-75%)				Preferred Practice Mean± SD Median (25%-75%)			
Statement	Governmental 1ry Healthcare center(n = 270)	Non-governmental hospital (n = 410)	University hospital (n = 412)	p-value	Governmental 1ry Healthcare center(n = 270)	Non-governmental hospital (n = 410)	University hospital (n = 412	p-value
“Helping patient/guardian decide”	5.7 ± 2.8	5.7 ± 2.7	4.4 ± 3.2	0.027*	5.2 ± 2.9	6.7 ± 2.7	5.4 ± 3.3	<0.001**
	5(3–8)	4(2–7)	4(2–7)		4(2–7)	6(3–8)	4(2–7)	
“Making sure patient/guardian understand	6.3 ± 2.7	6.0 ± 2.9	6.1 ± 2.9	0.006**	5.7 ± 2.9	5.4 ± 2.8	4.4 ± 2.9	0.006**
	6(3–8)	6(4–9)	6(5–8)		6(3–8)	4(2–7)	5(3–6)	
“Informing patient/guardian”	5.0 ± 2.8	4.5 ± 2.7	4.4 ± 2.7	0.616	6.1 ± 2.4	6.7 ± 2.9	6.8 ± 2.9	<0.001**
	5(2–7)	5(3–7)	5(3–7)		6(3–8)	4(2–6)	3(2–6)	
“Having shared decision”	5.5 ± 2.9	4.8 ± 2.8	4.7 ± 2.7	0.058	6.4 ± 3.0	6.3 ± 3.2	7.1 ± 3.0	0.006**
	5(2–7)	5(2–7)	5(3–7)		5(3–9)	6(4–9)	7(5–8)	
“Discovering patient’s preferences”	6.5 ± 2.8	6.2 ± 3.1	6.7 ± 3.4	<0.001*	5.5 ± 2.7	4.7 ± 2.7	4.2 ± 2.6	<0.001**
	4(2–6)	5(3–7)	6(4–8)		5(3–7)	5(4–7)	6(5–7)	
“Documenting patient’s/guardian decision”	4.8 ± 3.0	4.7 ± 2.7	5.0 ± 2.4	0.014*	5.5 ± 3.1	4.1 ± 2.4	4.1 ± 2.6	<0.001**
	6(3–8)	4(3–7)	4(2–7)		5(3–8)	5(2–6)	3(2–6)	
For litigation protection”	4.7 ± 2.7	4.9 ± 2.6	5.0 ± 2.5	<0.001**	4.7 ± 2.9	4.4 ± 2.6	5.0 ± 2.4	0.305
	6(4–8)	6(4–8)	4(1–8)		5(3–8)	6(3–7)	5(4–7)	
“Being a sort of courtesy gesture”	4.5 ± 2.8	5.2 ± 2.6	5.7 ± 2.5	0.000**	5.1 ± 2.8	5.2 ± 2.5	5.5 ± 2.4	<0.001**
	5(3–9)	8(5–10)	7(5–8)		5(3–8)	7(4–9)	6(1–8)	
“Taking away compensation rights”	5.7 ± 2.6	5.9 ± 2.8	6.4 ± 2.4	0.457	5.1 ± 2.6	5.5 ± 2.6	5.9 ± 2.3	0.002**
	7(4–9)	6 (4–9)	6(3–9)		6(4–8)	8(4–9)	8(4–9)	
“Being a sort of meaningless routine”	5.7 ± 3.0	7.0 ± 2.8	6.5 ± 2.6	0.012*	7.0 ± 2.8	6.0 ± 2.8	6.5 ± 2.4	<0.001**
	7(5–9)	6(4–9)	8(3–10)		7(4–9)	7(3–10)	8(4–10)	

SD: standard deviation. IQR: (25%-75%):

**highly sig,

*Significant P-value for Kruskal-Wallis test. Medians were used for the statistical test.

Table 4 showed the differences between patients/guardian perception of current (actual) and preferred practices of informed consent within each health facility. Two statements were found to be ranked significantly different in the current and preferred practice of informed consent in the three healthcare settings. These were “Making sure patients/guardians understand" (higher median rank in the preferred practice ranking) and “Having shared decision” (higher median rank in the current practice ranking). Three statements were found to be ranked significantly different between the current and preferred practice of informed consent in the university and non-governmental hospitals, namely: “Informing patients/guardians” (higher median rank in the preferred practice ranking), “Taking away compensation rights” and “for litigation protection” (higher median rank in the current practice).

**Table 4:** Differences between patients’ perception of the current and preferred purposes of the IC practices within each of the study facilities.

Statement	Governmental 1ry Healthcare center(n = 270)			Non-governmental hospital (n = 410)			University hospital (n = 412)		
	Current	Preferred	P-value	Current	Preferred	P-value	Current	Preferred	P-value
“Helping patient/guardian decide”	5(3–8)	4(2–7)	.044	4(2–7)	6(3–8)	< .001**	4(2–7)	4(2–7)	.793
“Making sure patient/guardian understand	6(3–8)	6(3–8)	< .001**	6(4–9)	4(2–7)	< .001**	6(5–8)	5(3–6)	< .001**
““Informing patient/guardian”	5(2–7)	6(3–8)	.053	5(3–7)	4(2–6)	< .001**	5(3–7)	3(2–6)	< .001**
“Having shared decision”	5(2–7)	5(3–9)	.006**	5(2–7)	6(4–9)	< .001**	5(3–7)	7(5–8)	< .001**
“Discovering patient’s/ guardian preferences”	4(2–6)	5(3–7)	.073	5(3–7)	5(4–7)	.133	6(4–8)	6(5–7)	.279
“Documenting patient’s decision”	6(3–8)	5(3–8)	.945	4(3–7)	5(2–6)	.416	4(2–7)	3(2–6)	.012*
For litigation protection”	6(4–8)	5(3–8)	.119	6(4–8)	6(3–7)	.015*	4(1–8)	5(4–7)	< .001**
“Being a sort of courtesy gesture”	5(3–9)	5(3–8)	.188	8(5–10)	7(4–9)	.052	7(5–8)	6(1–8)	< .001**
“Taking away compensation rights”	7(4–9)	6(4–8)	.360	6 (4–9)	8(4–9)	< .001**	6(3–9)	8(4–9)	< .001**
“Being a sort of meaningless routine”	7(5–9)	7(4–9)	.830	6(4–9)	7(3–10)	.668	8(3–10)	8(4–10)	.25

**highly sig,

*Significant P-value for Wilcoxon Signed ranks test. Medians rank were used for the statistical test.

Consensus agreement was found between the current and the preferred purposes of the IC within the three healthcare settings regarding: “Being a sort of meaningless routine” and “Discovering patient’s/ guardian preferences” which were reported by the majority to be ranked as the low purpose for IC practices.

Table 5 depicts in percentages the statements with the highest rank (1–5) in the three studied health facilities as perceived by patients for the current practice and preferred ones. The six statements with the best overall ranks (> 50%) considering the current practices were in order: “Helping patients/guardians decide (64.9%)”, “Documenting patient’s/guardian’s decision (59.3%)”, “Making shared decision (57.3%), Informing the patient/guardian (56.8%), Litigation protection (54.6%) and Discovering Patient/guardian preference (53.8%) These were ranked 1–5 by 64.9%, 59.3%, and 57.3% of respondents, respectively. The three statements with the best overall ranks considering the preferred practice were: “Informing the patient/guardian”, “Making sure patients understand” and “Documenting patient’s decision”. These were ranked 1–5 by 68.4%, 65.3%, and 65.1% of respondents, respectively with an insignificant difference (P>0.05) [Table 5].

**Table 5:** Comparison of percentages of high ranked perceived current and preferred patients’ purposes for the IC practices.

Statement	Current practice	Preferred practice
	Total	Total
Helping patient/guardian decide	64.9%	54.5%
Documenting patient’s/guardian’s decision	59.3%	65.1
Making shared decision	57.3%	Not highly ranked
Informing patient /guardian	56.8%	68.4%
For litigation protection	54.6%	51.4%
Making sure patient/guardian understand	Not highly ranked	65.3%
Discovering patient/guardian preference	53.8%	Not highly ranked

## Discussion

The study showed that IC implementation although highly implemented at more than two-thirds of different health care facilities, yet its implementation varied significantly (p<0.05) across the health care facilities in Egypt. Religious aspects could be the reason behind the IC high implementation for which the majority of the Egyptian community are Muslim and the minority are christened. The Religious aspects have shaped the Egyptian community’s opinions which were reflected in our study by the general agreement about the importance of the informed consent process with diversity in understanding its purpose [33]. The Committee of Senior Ulama (Arabic for religious scholars) explained the perception of some common rules based on the Quran and Sunna. For example, Quran encourages shared-decision making, it says, “And consult them in affairs.” (Chapter 3, verse 159). Quran also prohibits following others blindly without knowing their evidence (Chapter 33, verse 67; Chapter 43, verse 22; and Chapter 2, verse 111) [33]. Moreover, evidences from Quran and Sunna encourage taking good care of one’s body as well as seeking treatment (Chapter 16, verses 68–69 and Chapter 2, verse 195) [33], and Prophet Muhammad said, "There is no disease that Allah has created, except that He also has created its remedy." (Sahih al-Bukhari 5678) [34]. At the same time, Christian teachings also played a role in shaping the perception of Christian participants in the present study. Christian scripture encourages the practice of counseling in verses like “Seek counsel always of a wise man” (Tobit book, chapter 4, verse 19), Also informed consent is a practice which reflects honesty that is a value highly encouraged by Christian scripture in verses like “Open the gates that the righteous nation may enter, the nation that keeps faith” (Isiah book, chapter 26, verse 2).

The highest percentage of IC implementation was detected at the non-governmental facilities (85.9%) followed by the governmental facilities (77.8%), then at the university hospital (63.8). At the same time, the non-governmental (private) organization reported the highest percentage of giving enough information, while the university (Public teaching) hospital was reported the highest for not giving enough information. This difference was also significant indicating that the process in public hospitals looks like a document just to prevent the doctor and institution from possible future complaints in case of negative outcomes of the treatment. Hence, consent in its current form is not informed and should be re-evaluated to achieve patient autonomy. Similar findings were documented by many studies which reported that consent procedures appear inadequate in governmental hospitals and were more informative for the patient in private hospitals [35,36]. Another possible justification in the present Egyptian study could be related to the fact that most of the procedures experienced by the participants were elective procedures in which there is enough time to give enough information. However, the evidence from the systematic review done by Flynn et al. did not support the notion of accepting that an emergency procedure is an excuse for an incomplete or unsuccessful informed consent process [36]. Meanwhile, receiving “little or no information” was reported as the highest among university hospital participants. This could, at least in part, be explained by the fact that most of these participants were females who may be either neglected deliberately or not deliberately to be informed. This is also supported by an Egyptian study that promoted raising women’s awareness about their rights and considering it to be mandatory especially for rural communities [19]. Moreover, our explanation could be supported by the present study which showed a significant difference between both genders in the perception of the amount of information received in which a high percentage of females reported receiving “no enough information”.

Almost half of the participants in the present study were guardians of children and adolescents (45.79%) who are legally authorized for decision-making on behalf of their kids. As per the American Academy of Pediatrics, It is recommended to include children’s opinions in the decision-making process [5]. That recommendation is also supported by The National Council for Childhood and Motherhood in Egypt who emphasizes empowering all children whether their sex or background to say their opinions and share in decision-making in all aspects of life [37]. Moreover, the information given to children should be age-appropriate [38].

The present study showed that, out of the purposes of the current IC practice that were ranked 1–5 by respondents and were not highly ranked as preferred practices were perceived for making a shared decision” and “Discovering patient’s/ guardian preferences (ranked by 57.3% and 53.8% and of respondents, respectively). Meanwhile, the opposite was observed for “Making sure patient/guardian understand” which was ranked among the first five priorities as the preferred purpose for IC practices by 65.3% of respondents. Moreover, the “Making sure patient/guardian understand” statement was reported with a highly significant difference of preferred practice versus current practice. Such finding indicates the importance of respecting the right of the patients/ guardians to be not only informed but empowered with information especially if supported with audiovisual tools. This finding supports other studies which showed that patients require the doctor to inform them about all possible complications just to have a better idea of what to expect regardless of whether he is going to use this information to decide to undergo the procedure or not [39,40]. They highlighted the rights of all patients/guardians to be well informed about the benefits and risks, and that paternalistic assumptions were not acceptable [39,40]. This finding indicates that patients/guardians are caring about the quality and clarity of the information rather than about the amount of information received. This was supported by many community-based studies that were done for empowering women by information to have their rights and proved to be very successful in achieving a positive impact on health [19,26,41,42]. Furthermore, as reported in this study, written consent is the predominant tool for the informed consent process which is still the preferred method for pre-operative anesthetic counseling. Visual consent had the strongest influence on parents’ comprehension [43] and could hence be used to enhance the process of informed consent. Using multimedia tools help in lowering patient anxiety during the informed consent process [44]. Meanwhile, the majority of participants reported that they receive enough information during the consent process. Measuring the adequacy of information provided is one of the controversial issues. Many pieces of literature recommend the reasonable patient or guardian standard approach; which focuses on considering what the average patient or guardian would need to know in order to be an informed participant in the decision. This was found as the best approach in order to provide the best care to patients and to respect the patients’ rights to be involved in their health care decision [13,16,39,41,42]. However, patients still vary in the degree of details they want to know, so the surgeon should calibrate the information in accordance with the request [45]. In the majority of countries, the legal framework for consent requires guardian/parental approval and permission for young people aged below 18 years. Further, maintaining the role of parents as decision-makers for their child’s health care was frequently prioritized over enabling young people’s autonomy to consent [46,47].

This study showed that the three statements of “Helping patient/guardian decide”, “Documenting patients’/guardians’ decision”, and “Have shared decision” were ranked high by the majority of participants when ranking statements based on the current perceived purpose of informed consent. Also, the “Having shared decision” statement was reported with a highly significant difference of current practice versus preferred practice. Similar findings were presented in Ferrarese and his colleagues who reported that making a joint decision between patients or guardians and their physicians was more frequently reported compared to assigning more responsibility to the physician or giving patients or guardians full autonomy [12]. Physicians should involve pediatric patients in their health care decision-making by providing information on their illness and options for diagnosis and treatment in an appropriate manner with seeking assent to medical care [5].

Although consensus agreement was found between the current and the preferred purposes of the IC within the three healthcare settings regarding: “Being a sort of meaningless routine”, yet the majority of participants gave a low rank for the statement of a “meaningless routine” which indicates that they believe in the importance of the process of informed consent. Similar findings were reported by other studies as a minority of respondents perceived the clinical informed consent as a “being a sort of courtesy gesture” [31,48].

It is recommended that physicians must realize that IC, assent, or refusal constitutes a process, not a discrete event, and requires the sharing of information in ongoing physician-patient or family communication and education. This is also supported by many of the Egyptian studies about the effectiveness of increasing the awareness about their rights in receiving care especially among rural communities who always reported underutilization for the health services [16–20].

In conclusion, patients/guardians vary in their perception about the purpose of the informed consent. However, the majority of participants agreed about its importance and reported that they receive enough information during the informed consent process but with varied percentage according to the type of the health care setting.

The currently practiced consent process was perceived to be a decision making guiding process, while the participants’ preference is more toward the purpose of getting more information disclosure than involvement in decision-making. So additional information is recommended which includes but not limited to surgeon experiences and procedure expected outcomes with much more emphasis on making sure patient/guardian understand, documenting patient’s/guardian’s decision and informing patient /guardian rather than only sharing decision making. Actions to improve and standardize the content of informed consent are recommended for all categories of healthcare facilities.

As the dissatisfaction about amount of information received during informed consent process was mostly reported from participants interviewed in the university hospital, it may be indicated to prioritize improvement actions in this type of facility. It is also recommended to raise awareness among the healthcare providers for not underestimating the female rights of getting enough information during informed consent process and to empower females to seek their rights for receiving enough information.

As population expectations evolve with time, it is recommended to repeat and update that type of study which seeks patient opinions about important healthcare related matters such as informed consent process.

## Limitation of the study

Not all participants clearly understood the statements during the ranking process. However, such possible bias was minimized by providing an explanation to the participants by the attended study investigators prior to filling in the questionnaire. Fortunately, the consistency of responses observed among most of the participants could indicate that the final conclusion was not affected much by the assumed participant misunderstanding. Another important concern that according to the study aim and method, only patients’ experiences and perceptions were considered for assessment of the IC process. However, it may be helpful to consider third-party evaluation by observing healthcare providers- patients communication during the informed consent process to detect drawbacks if any and to raise practical recommendations to improve the process of informed consent.

### Strengths of the study

The study target population was matched with the topic as it is more relevant to assess the perception of clinical informed consent among persons who experienced the process in reality which supports the validity of the study conclusions. Also, testing the study hypothesis among the high number of participants with wide diversity attending more than one health care setting makings the findings more reliable and generalizable.

## Supporting information

S1 File Study questionnaire (English). Data description of the study questionnaire: Section I: participants’ demographics, procedure type and informed consent process data. Section II: Ten statements about potential purpose of informed consent process to be ranked considering participants’ perception of current purpose at the study institutions Section III: The ten statements about potential purpose of informed consent process in a random order to be ranked considering participants’ perception of preferred purpose. 1 is “most reflective” and 10 is “least reflective”.
(DOCX)

S2 File Informed consent raw data. (XLSX)

S3 File Informed consent raw data. (SAV)

## Abbreviations

IC: Informed consent
