# Supplementary material for: Egyptian patients’/guardians’ experiences and perception about clinical informed consent and its purpose: Cross sectional study
Source: PLoS One. 2021 Jun 14;16(6):e0252996. doi: 10.1371/journal.pone.0252996 (PMC8202917; doi:10.1371/journal.pone.0252996)
Supplement: S1 File — Data description of the study questionnaire: Section I: participants’ demographics, procedure type and informed consent process data. Section II: Ten statements about potential purpose of informed consent process to be ranked considering participants’ perception of current purpose at the study institutions Section III: The ten statements about potential purpose of informed consent process in a random order to be ranked considering participants’ perception of preferred purpose. 1 is “most reflective” and 10 is “least reflective”. (DOCX) [file pone.0252996.s001.docx]

**Participants in this questionnaire must have had a medical or surgical intervention within the past six months**

Name:

National ID:

Age:

Place:

Job:

Social status:

Number of family members:

Monthly household income:

Is the monthly income sufficient for family needs?

Type of procedure:

Location of procedure:

Date of procedure:

Was an informed consent given before the procedure?

Type of informed consent: Oral - Written

# Section one

Each of the following ten sentences describes a potential aim of informed written consent prior to medical interventions. Please rank the sentences from 1 to 10 to describe the extent to which they reflect the current situation in specialized hospitals. The sentence that is most representative of the current situation in the hospital where the procedure was performed should be given number "1". The sentence that least reflects the current situation should be given number "10". Each number should be used only once. These sentences are arranged randomly.

_______ Legal protection of the hospital and its staff.

_______ Prevent the patient from claiming compensation in case any of the complications listed in the consent happens.

_______ Help the patient make an informed decision regarding his or her health.

 _______ Document the decision made by the patient.

_______ Pointless, routine paperwork.

_______ Helps the patient and his doctor reach a shared decision.

_______ Inform the patient about what will happen or may happen during and after the procedure.

_______ Find out what the patient prefers and appreciates.

_______ Ensure that the patient is aware of and fully understands what will happen or may happen during and after the procedure.

_______ Gesture of courtesy or good treatment.

# Section two:

Each of the following 10 sentences describes a potential aim of informed written consent prior to medical interventions. Please rank the sentences from 1 to 10 according to what you think should take place in hospitals. The sentence that reflects the most what should be taking place in hospitals should be given number "1". The sentence that reflects the least what should be taking place in hospitals should be given number "10". The numbers 1 to 10 should be used only once. These sentences are arranged randomly.

_______ Ensure that the patient is aware of and fully understand what will happen or may happen during and after the procedure.

_______ Gesture of courtesy or good treatment.

_______ Pointless, routine paperwork.

_______ Help the patient make an informed decision regarding his or her health.

_______ Document the decision made by the patient.

_______ Inform the patient about what will happen or may happen during and after the procedure.

_______ Find out what the patient prefers and appreciates.

_______ Legal protection of the hospital and its staff.

_______ Prevent the patient from claiming compensation in case any of the complications listed in the consent takes place.

_______ Helps the patient and his doctor reach a shared decision.
